# Enzyme IIA^Ntr^ Regulates *Salmonella* Invasion Via 1,2-Propanediol And Propionate Catabolism

**DOI:** 10.1038/srep44827

**Published:** 2017-03-23

**Authors:** Woongjae Yoo, Dajeong Kim, Hyunjin Yoon, Sangryeol Ryu

**Affiliations:** 1 Department of Food and Animal Biotechnology, Department of Agricultural Biotechnology, and Research Institute for Agriculture and Life Sciences, Seoul National University, Seoul 08826, Korea; 2 Department of Molecular Science and Technology, Department of Applied Chemistry and Biological Engineering, Ajou University, Suwon 16499, Korea; 3 Institute of Food Industrialization, Institute of Green Bio Science & Technology, Seoul National University, Pyeongchang, Gangwon, Korea

## Abstract

Many *Proteobacteria* possess a nitrogen-metabolic phosphotransferase system (PTS^Ntr^) consisting of EI^Ntr^, NPr, and EIIA^Ntr^ (encoded by *ptsP, ptsO*, and *ptsN*, respectively). The PTS^Ntr^ plays diverse regulatory roles, but the substrate phosphorylated by EIIA^Ntr^ and its primary functions have not yet been identified. To comprehensively understand the roles of PTS^Ntr^ in *Salmonella* Typhimurium, we compared the whole transcriptomes of wild-type and a Δ*ptsN* mutant. Genome-wide RNA sequencing revealed that 3.5% of the annotated genes were up- or down-regulated by three-fold or more in the absence of EIIA^Ntr^. The Δ*ptsN* mutant significantly down-regulated the expression of genes involved in vitamin B_12_ synthesis, 1,2-propanediol utilization, and propionate catabolism. Moreover, the invasiveness of the Δ*ptsN* mutant increased about 5-fold when 1,2-propanediol or propionate was added, which was attributable to the increased stability of HilD, the transcriptional regulator of *Salmonella* pathogenicity island-1. Interestingly, an abundance of 1,2-propanediol or propionate promoted the production of EIIA^Ntr^, suggesting the possibility of a positive feedback loop between EIIA^Ntr^ and two catabolic pathways. These results demonstrate that EIIA^Ntr^ is a key factor for the utilization of 1,2-propanediol and propionate as carbon and energy sources, and thereby modulates the invasiveness of *Salmonella* via 1,2-propanediol or propionate catabolism.

Most *Proteobacteria* possess the regulatory nitrogen-metabolic phosphotransferase system (PTS^Ntr^), which operates in parallel with the phosphoenolpyruvate (PEP)-dependent carbohydrate PTS^1^,^2^,^3^. PTS^Ntr^ is composed of the proteins EI^Ntr^ (encoded by *ptsP*) and NPr (encoded by *ptsO*) and the final phosphate acceptor EIIA^Ntr^ (encoded by *ptsN*). In this system, three proteins form a phosphorylation chain working in the sequential order EI^Ntr^ → NPr → EIIA^Ntr^^4^,^5^ (Supplementary Fig. S1). EIIA^Ntr^ is known to play various regulatory roles relevant to potassium (K^+^) transport^6^,^7^, phosphate homeostasis^8^, σ factor selectivity^9^, fluxes through carbohydrate pathways and central metabolism^10^,^11^, stringent response^12^,^13^, and virulence^14^,^15^. For instance, EIIA^Ntr^ maintains intracellular K^+^ homeostasis by binding to the low-affinity K^+^ transporter TrkA and the sensor histidine kinase KdpD in the high-affinity K^+^ transporting system in *Escherichia coli*^6^,^7^, which results in K^+^-mediated global gene regulation in association with both σ^D^- and σ^S^-dependent promoters^16^. Dephosphorylated EIIA^Ntr^ is required for full adaptation to phosphate limitation conditions through direct interaction with the sensor kinase PhoR in a two-component system involved in inorganic phosphate homeostasis^8^, and it also controls the connections between C metabolism and many other cellular functions by binding to pyruvate dehydrogenase (PDH), which generates acetyl-CoA from pyruvate^17^. It has recently been revealed that, in *Caulobacter crescentus*, EIIA^Ntr^ inhibits the hydrolase activity of SpoT by direct interaction^13^, which influences the cellular accumulation of (p)ppGpp, an alarmone controlling bacterial cell cycle progression, growth, and virulence, for its adaptation to environmental changes. In addition, EIIA^Ntr^ is associated with virulence in *Legionella pneumophila*^15^ and *Salmonella enterica*^14^.

*Salmonella enterica* serovar Typhimurium is a bacterial pathogen that can infect a wide range of animals and causes food-borne gastroenteritis in millions of people worldwide^18^,^19^. In an animal intestinal tract, *Salmonella* competes with the resident bacteria occupying colonization niches, and it has evolved to harness diverse metabolic pathways to enhance its fitness during infection^20^. For instance, *Salmonella* can acquire carbon sources from 1,2-propanediol (1,2-PDL), an abundant fermentation product derived from the plant sugars L-rhamnose and L-fucose. 1,2-PDL is degraded into propionaldehyde by vitamin B_12_-dependent 1,2-propanediol dehydratase and further processed to 1-propanol and propionate with the coordinated action of *pdu* operon products^21^,^22^ (Fig. 1). In animal intestines, propionate is also provided at a high concentration as a fermentation byproduct of microflora^23^. *Salmonella* with the *prp* operon converts propionate to propionyl-Coenzyme A (propionyl-CoA) and further degrades into pyruvate and succinate via the 2-methylcitric acid cycle (MCC)^24^,^25^ (Fig. 1). Propionyl-CoA is therefore known as a common intermediate linking the 1,2-PDL and propionate catabolism circuits^26^.

In addition to being a metabolic intermediate for energy production, propionyl-CoA serves as a regulatory signal compromising the stability of HilD, the transcriptional regulator of *Salmonella* pathogenicity island-1 (SPI-1). SPI-1, a chromosomal region composed of 39 genes, encodes a type 3 secretion system (T3SS), whereby its cognate effector proteins are translocated into host cells to promote bacterial invasion. An increase in propionyl-CoA production compromises the stability and activity of HilD in a post-translational manner, thus attenuating *Salmonella* invasion into intestinal epithelial cells^27^. Although SPI-1 is prominently responsible for *Salmonella* invasion into host cells, SPI-4 is also required for bacterial adhesion to polarized epithelial cells as well as for invasion^28^. The SPI-4 locus contains six genes, *siiABCDEF*, forming an operon. SiiE, which is secreted via an SPI-4-encoded type 1 secretion system (SPI-4 T1SS), facilitates *Salmonella* adhesion to polarized host cells. Not surprisingly, the expression of SPI-4 is coordinated with SPI-1 regulation. SPI-1 uses its cognate regulators (HilD, HilC, HilA, and InvF) for its systematic regulation. HilD and HilC induce the expression of HilA in combination or independently. HilA, in turn, directly activates the expression of *invF* and the genes encoding T3SS and also indirectly induces the transcription of SPI-4. HilA antagonizes the H-NS-mediated transcriptional silencing of SPI-4^29^.

To better understand the roles of EIIA^Ntr^, we used RNA-seq to compare the whole transcriptomes of the wild-type and a mutant strain lacking *ptsN*. Genes involved in vitamin B_12_ synthesis, 1,2-PDL utilization, and propionate catabolism were significantly down-regulated in the Δ*ptsN* mutant strain, whereas SPI-1 and SPI-4 were up-regulated. In accordance with the transcriptional differences, the Δ*ptsN* mutant strain was more competent in invasion into host cells than the wild-type bacteria when they were pre-cultured with 1,2-PDL or propionate. We found that EIIA^Ntr^ can control *Salmonella* invasion by balancing propionyl-CoA in response to environmental carbon metabolites.

## Results

### Comprehensive understanding of the roles of EIIA^Ntr^ in *Salmonella* using transcriptome analysis

EIIA^Ntr^ plays multifaceted regulatory roles in diverse bacterial species, including *Escherichia coli*^6^,^7^, *Pseudomonas putida*^30^ and *Salmonella* Typhimurium^14^. In this study, transcriptome profiling of a *Salmonella* Δ*ptsN* mutant strain was conducted to understand the comprehensive roles of EIIA^Ntr^ in *Salmonella* metabolism and virulence. The Δ*ptsN* mutant strain did not show growth defects in LB broth at 37 °C compared to its parent strain, *S.* Typhimurium SL1344 (Supplementary Fig. S2a and S2b). Total RNA was extracted at a mid-exponential phase of growth (OD_600_ = 1) and analyzed using RNA-seq. Mapping analysis of RNA-seq data was summarized in Supplementary Table S1. A statistical comparison between the two strains (p value < 0.05) revealed that 2.40% (116/4837) and 1.12% (54/4837) of the total genes were up- and down-regulated, respectively, by 3-fold or more in the Δ*ptsN* mutant strain compared to the wild-type (Fig. 2a). The differentially expressed genes (DEGs) were categorized based on their predicted functions by cluster of orthologous group designations^31^. Genes with functions in energy production and conversion, amino acid transport and metabolism, coenzyme transport and metabolism, and secondary metabolites biosynthesis/transport/catabolism were down-regulated overall in the absence of EIIA^Ntr^, and genes involved in cell wall/membrane/envelope biogenesis, inorganic ion transport and metabolism, signal transduction, intracellular trafficking/secretion/vesicular transport, and defense mechanisms were generally up-regulated (Fig. 2b). The likelihood of multiphasic cellular regulation by EIIA^Ntr^ was predicted from the previously identified roles of EIIA^Ntr^ in K^+^ homeostasis^6^,^7^, phosphate starvation responses^8^, ppGpp synthesis/hydrolysis^12^, amino acid synthesis/metabolism^32^,^33^, and carbon metabolism^17^. Taken together, these results suggest that EIIA^Ntr^ affects diverse metabolisms associated with *Salmonella* fitness.

### EIIA^Ntr^ activated expression of genes involved in 1,2-PDL utilization and propionate catabolism

**Figure 1:**
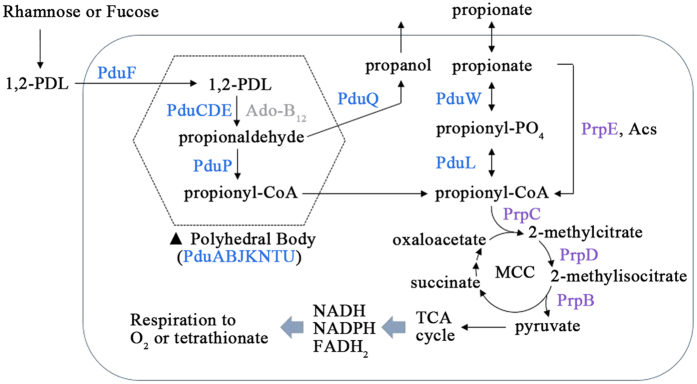
Catabolism of 1,2-PDL and propionate in S. Typhimurium. 1,2-PDL catabolism is achieved by the 1,2-PDL utilization operon, indicated in blue. 1,2-PDL is converted into propionyl-CoA within the polyhedral microcompartment. Propionate catabolism is accomplished by the propionate operon, indicated in purple. The common intermediate propionyl-CoA provides pyruvate through the 2-methylcitrate cycle (MCC), which can be used as an energy source. Oxygen or tetrathionate is used as an electron acceptor under aerobic or anaerobic conditions, respectively.

**Figure 2:**
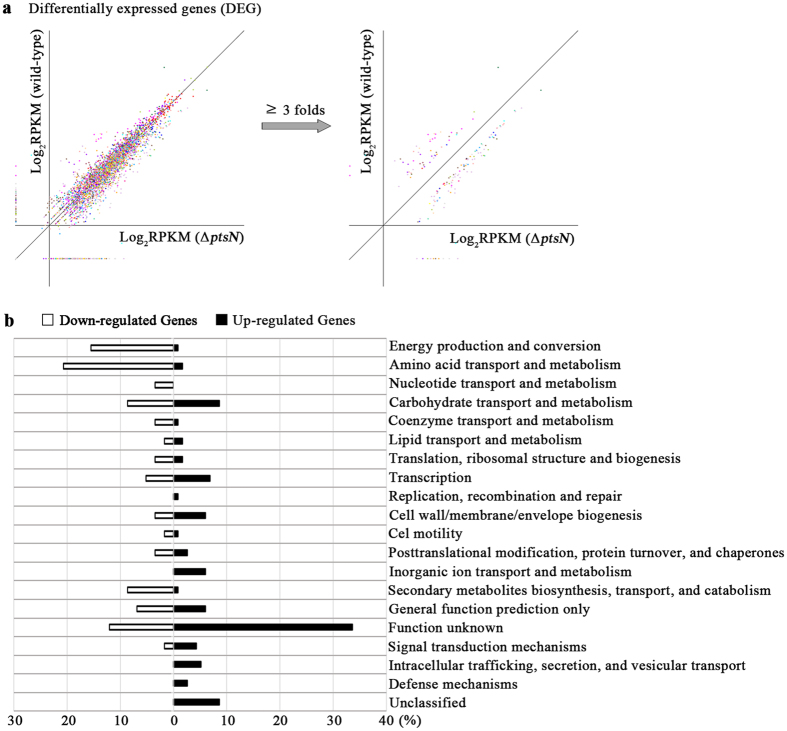
Classification of differentially expressed genes (DEGs) based on predicted functions. (**a**) DEG analysis from RNA-seq data between wild-type *Salmonella* and the Δ*ptsN* mutant strain. The y-axis shows the log-scaled RPKM values of wild-type, and the x-axis shows the log-scaled RPKM values of the Δ*ptsN* mutant strain. Total gene expressions in the two strains (left) were filtered to sort significantly down- or up-regulated genes (right) with the criteria of p-value ≤ 0.05 and fold-change ≥3. (**b**) Genes up-regulated or down-regulated by 3-fold or more in the Δ*ptsN* mutant strain are grouped into functional categories. Genes are annotated based on the cluster of orthologous groups database.

**Figure 3:**
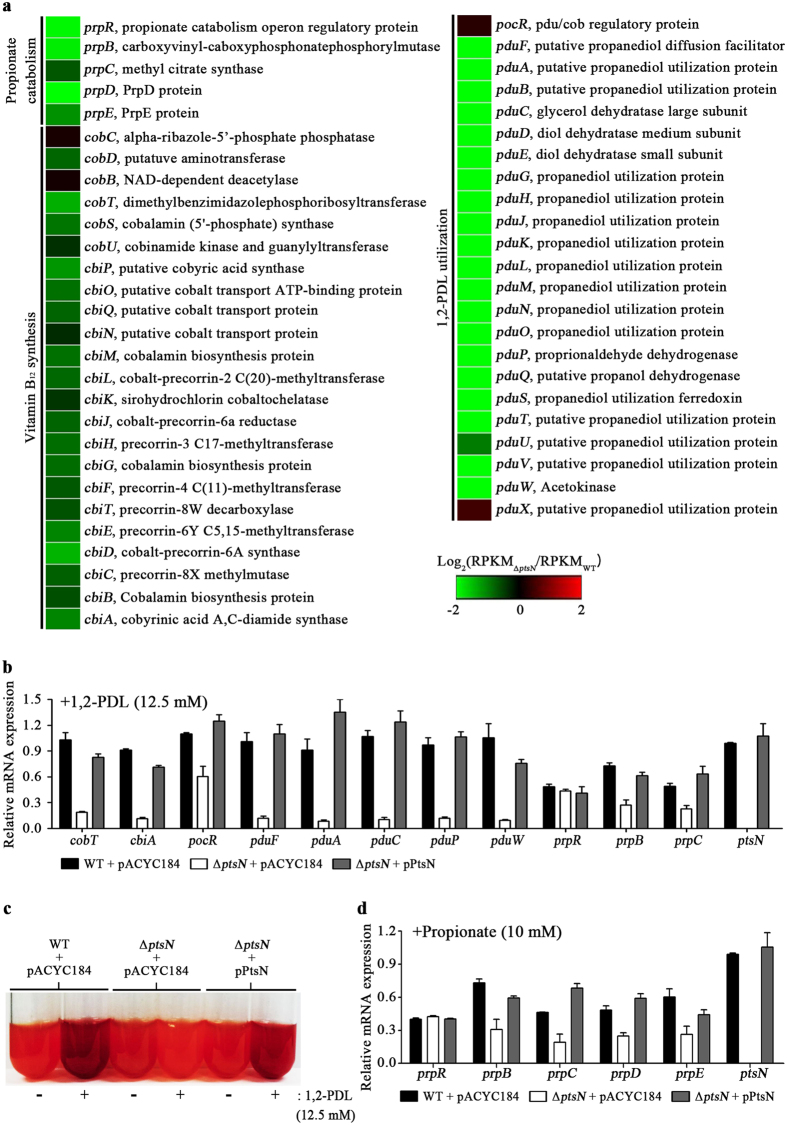
The effects of *ptsN* on the expression of genes involved in propionate catabolism, vitamin B_12_ synthesis, and 1,2-PDL utilization. (**a**) Heat-map analysis of the expression ratios of the *prp, cob-cbi*, and *pdu* genes between the Δ*ptsN* mutant and wild-type strains (log_2_[Δ*ptsN* mutant/wild-type]). (**b**) Evaluation of mRNA levels of the *cob-cbi, pdu*, and *prp* genes in strains of wild-type, Δ*ptsN*, and Δ*ptsN* containing pPtsN. Every mRNA level was normalized using that of *gyrB* in each strain and its relative expression was estimated comparing with the mRNA level of *ptsN* in wild-type containing pACYC184, which was set at 1.0. The pPtsN plasmid has *ptsN* and its putative promoter sequences on the backbone of pACYC184. Ado-B_12_ (20 nM) and 1,2-PDL (12.5 mM) were added to the culture to stimulate the *prp* operon under aerobic conditions. (**c**) Medium pH was assayed to compare the activity of 1,2-PDL catabolism in wild-type SL1344 containing pACYC184 and the Δ*ptsN* mutant strain harboring pPtsN or pACYC184. Neutral-red (0.0033%) was added to the culture as a pH indicator and changed its color to dark red in proportion to the level of propionate, a byproduct of the 1,2-PDL pathway. All cultures were supplemented with Ado-B_12_ (20 nM), and 1,2-PDL (12.5 mM) was added as a substrate for the 1,2-PDL pathway. The result shown is representative of three independent tests. (**d**) The mRNA levels of *prp* genes were measured in a similar manner as conducted in (**b**) with normalization to the mRNA level of *ptsN* in the wild-type containing pACYC184, but propionate (10 mM) was added as an alternative carbon source instead of 1,2-PDL and Ado-B_12_.

An effort to make sense of the primary roles of EIIA^Ntr^ in *Salmonella* led to a search for genes coordinately controlled as an operon by EIIA^Ntr^. Genes constituting the 1,2-PDL utilization (*pdu*) operon mostly decreased in the Δ*ptsN* mutant strain, indicating a positive role of EIIA^Ntr^ in utilizing 1,2-PDL as a carbon source (Fig. 3a; Supplementary Table S2). The *pdu* operon consists of 21 genes, with *pocR* and *pduF* encoding its cognate activator and membrane transporter, respectively, in the opposite direction on the chromosome and encodes proteins for the catabolism of 1,2-PDL. *Salmonella* degrades 1,2-PDL into propionaldehyde with the aid of the cofactor adenosyl cobalamin (Ado-B_12_) and further processes it into propanol and propionate through propionyl-CoA to acquire energy in nutrient-restricted conditions^22^. In accordance with a decrease in *pdu* expression, the *cob-cbi* operon encoding enzymes for the biosynthesis of Ado-B_12_ was inclined toward down-regulation by the absence of EIIA^Ntr^ (Fig. 3a; Supplementary Table S2). Propionyl-CoA is a common intermediate linking the 1,2-PDL and propionate catabolic pathways (Fig. 1). Propionyl-CoA, produced in the middle of the 1,2-PDL degradation pathway or synthesized from propionate by multiple propionyl-CoA-synthesizing systems, is integrated into the MCC and further catabolized to pyruvate and succinate through the coordinated action of *prp* operon-encoded enzymes^24^,^25^. Thus, the 1,2-PDL and propionate catabolic pathways enable *Salmonella* to produce energy and carbon sources and outcompete commensal microbiota in a nutrient-restricted host intestine^20^. The transcriptome profiling revealed that four genes of the *prp* operon and their regulator gene *prpR*, which are transcribed divergently on the chromosome, were down-regulated in step with the *pdu* and *cob-cbi* operons in the Δ*ptsN* mutant strain (Fig. 3a; Supplementary Table S2), indicating that EIIA^Ntr^ coordinately activates transcription of multiple genes to exploit 1,2-PDL and propionate, which are disfavored carbon sources for the bystander microbiota^20^. The levels of mRNAs relevant to vitamin B_12_ synthesis (*cobT* and *cbiA*), 1,2-PDL utilization (*pocR, pduF, pduA, pduC, pduP*, and *pduW*), and propionate catabolism (*prpB* and *prpC*) were measured using qRT-PCR to verify the RNA-seq results. The lack of EIIA^Ntr^ decreased the expression of genes involved in vitamin B_12_ synthesis and 1,2-PDL and propionate catabolic pathways in accordance with the transcriptome analysis (data not shown). As expected, when *Salmonella* strains were supplemented with 1,2-PDL and Ado-B_12_, genes of the *cob-cbi, pdu*, and *prp* operons were much less transcribed in the Δ*ptsN* mutant strain than in the wild-type, and those decreases were complemented with *trans*-encoded EIIA^Ntr^ by the pPtsN plasmid (Fig. 3b).

### EIIA^Ntr^ negatively controls expression of SPI-1 and SPI-4, which are important for *Salmonella* invasion

**Figure 4:**
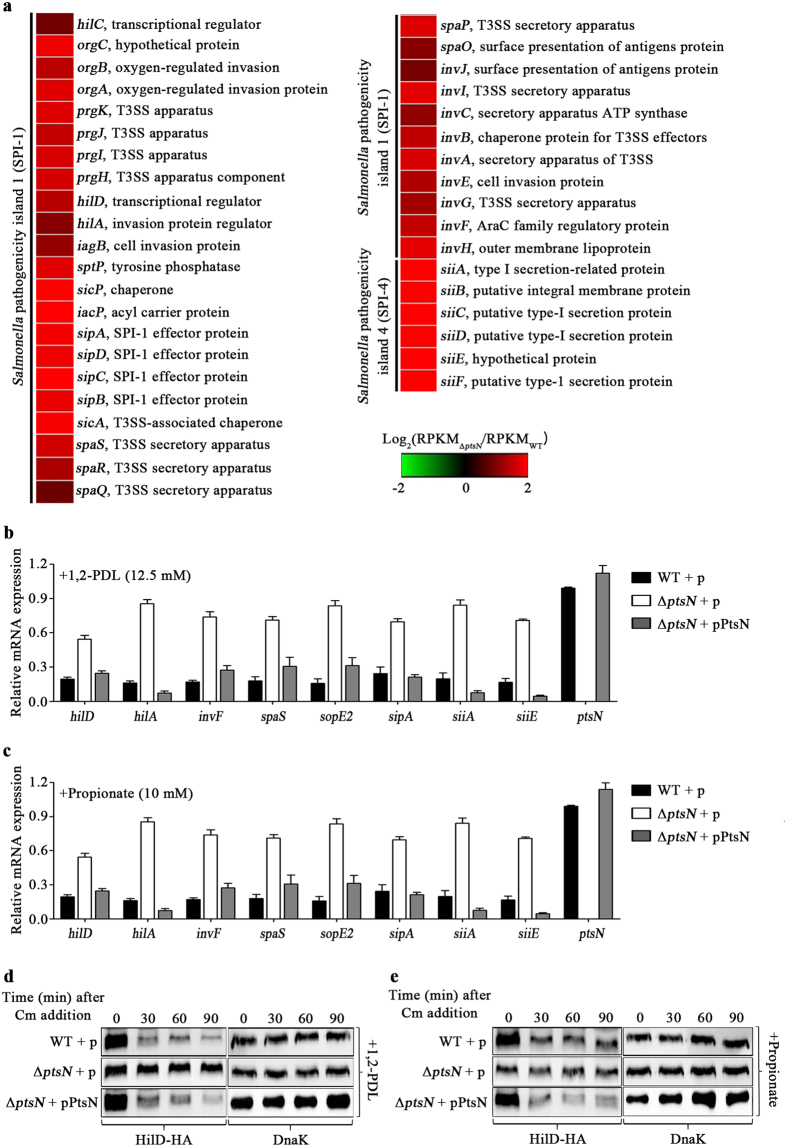
The effects of ptsN on the expression of genes involved in bacterial invasiveness. (**a**) Heat-maps generated to describe the expression patterns of SPI-1 and SPI-4 genes in the Δ*ptsN* mutant strain. Gene expression ratios between the Δ*ptsN* mutant and wild-type SL1344 are shown using a colorimetric gradient: down-regulation in green and up-regulation in red. (**b**,**c**) The relative mRNA levels of the genes involved in SPI-1 and SPI-4, as measured by qRT-PCR. The tested genes are the SPI-1 regulator genes (*hilD, hilA*, and *invF*), SPI-1 genes (*spaS, sopE2*, and *sipA*), and SPI-4 genes (*siiA* and *siiE*). As alternative carbon sources, Ado-B_12_ (20 nM), 1,2-PDL (12.5 mM), and propionate (10 mM) were added to the cultures of (**b** and **c**), respectively. The mRNA level of each gene was normalized using that of *gyrB* in the wild-type strain containing pACYC184 and the Δ*ptsN* mutant strains with either pACYC184 or pPtsN and its relative expression was calculated using the mRNA level of *ptsN* in the wild-type containing pACYC184, which was set at 1.0. All experiments were performed in triplicate, and the average values are depicted in the graphs. (**d** and **e**) The stability of the HilD-HA protein, compared under conditions identical to those used in (**b**) and (**c**). Three strains of wild-type with pACYC184 and Δ*ptsN* mutant strains with either pACYC184 or pPtsN were cultivated in the presence of 1,2-PDL (**d**) or propionate (**e**). *De novo* protein synthesis was quenched by the addition of chloramphenicol (0.2 mg/ml) at 4 h post inoculation. Total proteins were harvested every 15 min, and equivalent amounts were subjected to SDS-PAGE analysis. The stability of HilD-HA was assessed using anti-HA antibody. The levels of DnaK as controls were comparable between lanes. Full-length blots were presented in Supplementary Figure S5.

**Figure 5:**
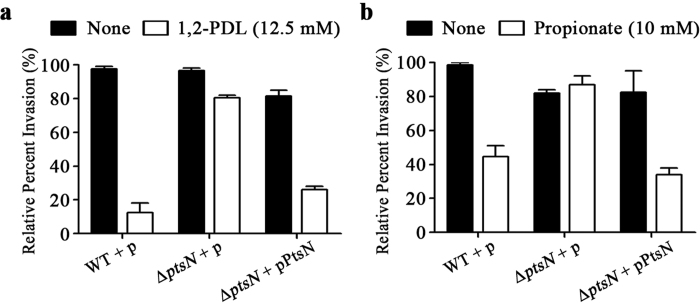
Invasion of epithelial cells by *Salmonella* in the presence of 1,2-PDL or propionate. (**a**) Three *S.* Typhimurium strains, wild-type, the Δ*ptsN* mutant, and the Δ*ptsN* mutant containing pPtsN, were pre-cultured with Ado-B_12_ (20 nM) and 1,2-PDL (12.5 mM) or not prior to infection and added to monolayers of Caco-2 cells at an MOI of 10. At 1.5 h post infection, cells were lysed, and intracellular bacteria were enumerated by plating. Values represent the relative amount of internalized bacteria and are normalized to the level of internalization of the wild-type containing the empty vector, which was set at 100%. (**b**) A similar invasion test was conducted, but propionate (10 mM) was provided as an alternative carbon source pre-cultivation. All invasion tests were carried out independently at least three times, and the averaged values are shown with standard deviations.

Recently, propionyl-CoA, the intermediate in the 1,2-PDL and propionate catabolic pathways, was found to function as a regulatory signal. Intracellular propionyl-CoA synthesized from the propionate abundant in the host intestine attenuated the stability and activity of HilD, the master regulator of SPI-1, which led to reduced *Salmonella* invasion into epithelial cells^27^. In agreement with that observation, altered expression of the *pdu* and *prp* operons, which depends on the presence of EIIA^Ntr^, also influenced the expression of SPI-1 and SPI-4 (Fig. 4a; Supplementary Table S2). The Δ*ptsN* mutant strain more weakly degraded 1,2-PDL into propionyl-CoA and propionate than did wild-type *Salmonella*, turning the neutral-red, an acidic pH indicator, light red instead of dark red like the wild-type strain (Fig. 3c). A reduction in propionyl-CoA in the Δ*ptsN* mutant strain presumably activates SPI-1 expression through the accumulation of HilD, which in turn stimulates SPI-4 expression via HilA. Differential expression of the SPI-1 and SPI-4 genes depending on the presence of EIIA^Ntr^ was also validated using qRT-PCR (Fig. 4b and c). When 1,2-PDL or propionate was added to the culture for conversion into propionyl-CoA, the Δ*ptsN* mutant strain increased SPI-1 and SPI-4 expression, and that altered expression was reversed by the introduction of pPtsN, producing EIIA^Ntr^ *in trans*. Thus, the capacity to use 1,2-PDL and propionate as carbon sources influences *Salmonella* invasiveness into epithelial cells (Fig. 5). The presence of 1,2-PDL and propionate attenuated the invasiveness of *Salmonella* wild-type strains, probably through an increase in propionyl-CoA via the 1,2-PDL and propionate catabolic pathways, whereas the differential invasion ability in response to 1,2-PDL and propionate was abolished in the absence of EIIA^Ntr^. Complementing the Δ*ptsN* mutant strain with pPtsN producing EIIA^Ntr^ *in trans* restored the ability to control invasiveness depending on the presence of 1,2-PDL or propionate. Consequently, this result suggests that EIIA^Ntr^ influences the ability of *Salmonella* to invade into host cells by manipulating propionyl-CoA production when 1,2-PDL or propionate is abundant.

### Dissecting EIIA^Ntr^ roles in transcriptional regulation of *pdu* and *prp* operons and SPI-1 and -4

The 1,2-PDL and propionate catabolic pathways are tightly linked by their use of propionyl-CoA as a common intermediate. The two pathways together accomplish the conversion between propionate and propionyl-CoA (Fig. 1). In this context, the influence of EIIA^Ntr^ on *prp* expression might be indirect, mediated by the activity of the 1,2-PDL pathway discharging propionyl-CoA and propionate into the propionate pathway. The addition of 1,2-PDL led to increases in *prp* and *pdu* expression, and the lack of EIIA^Ntr^ diminished expression of both *prp* and *pdu* (Fig. 3b and Supplementary Fig. S3b). On the other hand, the addition of propionate activated *prp* genes but not *pdu* genes (Fig. 3d and Supplementary Fig. S3). Furthermore, the *prp* operon was activated in response to propionate even when the 1,2-PDL pathway was blocked (Supplementary Fig. S3b), indicating the possibility of diverting the propionate pathway from 1,2-PDL depending on available carbon sources. The positive role of EIIA^Ntr^ in *prp* operon expression was also achieved in propionate-supplemented conditions, where the 1,2-PDL pathway was suspended (Fig. 3d and Supplementary Fig. S3b). Thus, EIIA^Ntr^ likely controls the 1,2-PDL and propionate catabolic pathways in a pleiotropic manner in response to carbon sources: coordinated activation of two pathways in the presence of 1,2-PDL and localized activation of the propionate pathway in the presence of propionate only.

**Figure 6:**
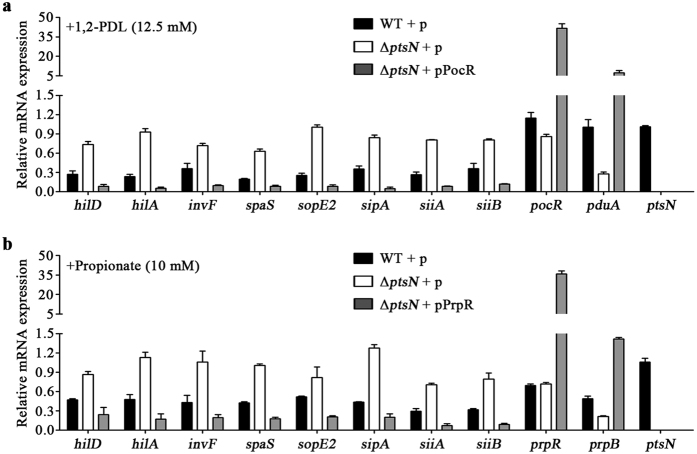
The effects of PocR or PrpR overexpression on SPI-1 and SPI-4 in the Δ*ptsN* mutant strain in the presence of 1,2-PDL or propionate. (**a**) The relative mRNA expression of genes involved in SPI-1 and SPI-4 was determined by qRT-PCR in the wild-type containing pUHE21-2*lacI*^q^ and in the Δ*ptsN* mutant strains harboring either pUHE21-2*lacI*^q^ or pPocR. *pocR*, which encodes the transcriptional activator for the *pdu* operon, was induced by the addition of 1 mM IPTG from pPocR. Strains were grown in LB medium containing 1,2-PDL (12.5 mM) as an additional carbon source. (**b**) A similar qRT-PCR was performed, but pPrpR producing PrpR, the transcriptional activator of the *prp* operon, was used instead, and propionate (10 mM) was added accordingly. Every mRNA level was normalized using that of *gyrB* and its relative expression to *ptsN* was averaged from three assays at least.

Propionyl-CoA is known to reduce *Salmonella* SPI-1 expression by destabilizing HilD and lowering its activity post-translationally^27^. SPI-1 expression is tightly controlled by a multitude of regulators, including HilD, HilC, RtsA, HilA, and InvF, though HilD holds predominant control of the regulatory circuit^34^,^35^. SPI-4, which is activated by HilA, is coupled with SPI-1 in the context of transcriptional regulation and cellular function and stimulates *Salmonella* invasion into host cells in concert with SPI-1^29^. Therefore, the increased expression of SPI-1 and SPI-4 in the Δ*ptsN* mutant strain was likely attributable to the HilD that accumulated at low propionyl-CoA levels. To examine this possibility, the stability of the HilD protein was compared between the wild-type and Δ*ptsN* mutant strains in the presence of 1,2-PDL or propionate as a precursor for propionyl-CoA. The Δ*ptsN* mutant strain maintained HilD continually for at least 90 min after chloramphenicol addition, whereas wild-type *Salmonella* degraded HilD gradually (Fig. 4d and e). Furthermore, the negative role of EIIA^Ntr^ in SPI-1 and SPI-4 expression was reversible by the overexpression of PocR and PrpR, the cognate activators for the *pdu* and *prp* operons, respectively (Fig. 6a and b). These results suggest that EIIA^Ntr^ affects the expression of SPI-1 and SPI-4 indirectly by modulating HilD post-translationally via the 1,2-PDL and propionate catabolic pathways.

### Translational levels of EIIA^Ntr^ are up-regulated in response to 1,2-PDL and propionate

**Figure 7:**
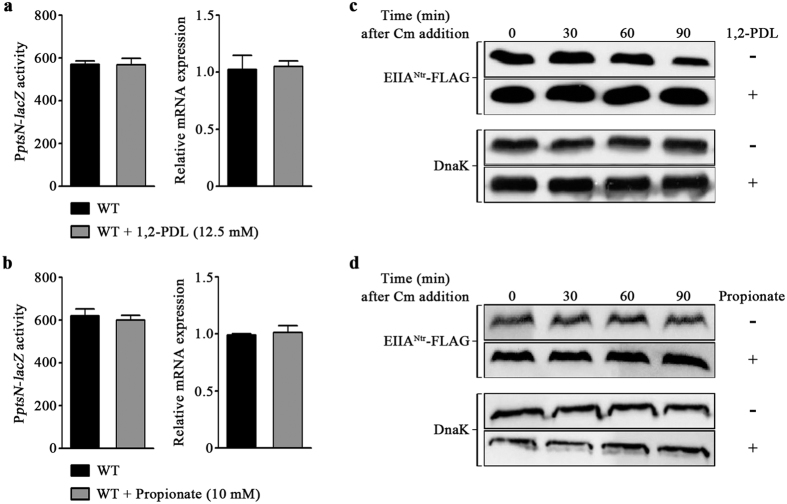
EIIA^Ntr^ production increased in response to 1,2-PDL and propionate. (**a** and **b**) The transcriptional expression of *ptsN* was measured with or without 1,2-PDL (**a**) and propionate (**b**). β-Galactosidase activity from P*ptsN*::*lacZ* was measured in Miller units (left), and the relative *ptsN* mRNA expression was determined using qRT-PCR (right). Each bar represents the average value of three independent experiments. (**c** and **d**) The stability of EIIA^Ntr^-FLAG was evaluated by western blot analysis with or without 1,2-PDL (12.5 mM) (**c**) and propionate (10 mM) (**d**). At 5 h post inoculation, mRNA translation was halted by adding chloramphenicol (0.2 mg/ml), and that time was set as 0 min. Total proteins were harvested every 30 min, and equivalent amounts were subjected to SDS-PAGE analysis. The stability of EIIA^Ntr^-FLAG was assessed using an anti-FLAG antibody. The levels of DnaK were used as controls. Full-length blots were presented in Supplementary Figure S6.

*ptsN* is a component of the *rpoN* operon, which is co-transcribed with *rpoN* from a single promoter upstream of *rpoN*. Because *rpoN-*encoded sigma 54 (σ^54^) controls the transcription of a plethora of genes involved in nitrogen assimilation and stress responses, PTS^Ntr^ has been considered as a regulatory system associated with nitrogen metabolism^2^,^3^. However, it remains unclear which stimuli trigger the activation of PTS^Ntr^. Intracellular balance between nitrogen and carbon was found to modulate the phosphorylation status of EIIA^Ntr^, the output regulator of PTS^Ntr^^36^. In addition, the availability of amino sugar was recently revealed to control the degradation rate of EIIA^Ntr^^37^. The finding that the positive role of EIIA^Ntr^ in *pdu* or *prp* expression is remarkable only in the presence of 1,2-PDL or propionate prompted analysis of whether the expression or activity of EIIA^Ntr^ could be enhanced in response to 1,2-PDL or propionate. The expression level of *ptsN* was measured in two different ways: a β-gal assay using *lacZ* fusion to the promoter of *ptsN* and *ptsN* mRNA quantification using qRT-PCR. The transcription of *ptsN* was unaffected by 1,2-PDL or propionate (Fig. 7a and b). However, the protein level of EIIA^Ntr^ was significantly higher in the presence of 1,2-PDL (Fig. 7c) or propionate (Fig. 7d). To rule out the possibility that the different EIIA^Ntr^ levels were caused by accelerated degradation of EIIA^Ntr^ in the absence of 1,2-PDL or propionate, the stability of EIIA^Ntr^ was compared after quenching protein synthesis using chloramphenicol. Degradation rates of EIIA^Ntr^ were not influenced by the presence of 1,2-PDL or propionate, showing comparable protein levels for 90 min (Fig. 7c and d). Thus, *ptsN* mRNA might be translated into EIIA^Ntr^ with different efficiency depending on the abundance of 1,2-PDL or propionate.

## Discussion

In this study, we found that *ptsN-*encoded EIIA^Ntr^, a component of the nitrogen-metabolic PTS, is a key factor controlling vitamin B_12_ synthesis, 1,2-PDL utilization, propionate catabolism, and invasion in *S.* Typhimurium. Transcriptome analysis using RNA-seq revealed that a lack of EIIA^Ntr^ decreased the expression of genes required for vitamin B_12_ synthesis, 1,2-PDL utilization, and propionate catabolism, and we validated that observation by qRT-PCR using total RNA extracted from cultures grown in the presence of 1,2-PDL or propionate. In accordance with a previous report about the negative regulation of SPI-1 by propionyl-CoA^27^, we found that the down-regulation of the 1,2-PDL and propionate pathways in the Δ*ptsN* mutant strain alleviated the rapid degradation of HilD, a dominant SPI-1 activator, and consequently led to an increase in invasion ability of *Salmonella*.

EIIA^Ntr^ has been reported to exert its regulatory activity via direct protein-protein interactions^6^,^7^,^8^,^13^,^14^. In an effort to understand how EIIA^Ntr^ orchestrates 1,2-PDL and propionate catabolism, we examined the possibility of direct protein-protein interaction between EIIA^Ntr^ and various regulatory factors associated with both catabolism using bacterial two-hybrid system^38^. Four different proteins including regulators of 1,2-PDL and propionate operons (PocR and PrpR, respectively), cAMP receptor protein (CRP), and adenylyl cyclase (AC) were tested but none of them showed interaction with EIIA^Ntr^ (unpublished data). CRP and AC are involved in the regulation of cobalamin regulon as well as 1,2-PDL and propionate operons^39^,^40^. However, it cannot be ruled out that other regulatory factors might coordinate the interaction between EIIA^Ntr^ and 1,2-PDL or propionate catabolism directly or indirectly.

*S.* Typhimurium causes non-typhoid gastroenteritis in humans and typhoid-like disease in mice^41^,^42^. During infection, *Salmonella* invades intestinal epithelial cells and replicates inside macrophages^43^,^44^. The ability to acquire nutrients during the infection process is crucial for enteric pathogens to survive and proliferate in the host because they have to compete with commensal microbiota for limited nutrients^20^. With regard to bacterial competition for restricted carbon sources, *S.* Typhimurium outcompetes commensal bacteria through two distinctive but closely linked pathways: the 1,2-PDL utilization pathway and the propionate catabolic pathway^20^. Plant sugars such as L-rhamnose and L-fucose, which are abundant in digested food molecules, are degraded into 1,2-PDL by gut microbiota. L-fucose constitutes mucosal glycoconjugates as a terminal sugar of the oligosaccharide chains linked to the mucin protein backbone and is easily accessible to enteric bacteria in the intestinal lumen^22^,^45^. Propionate is also provided at high concentrations through several fermentation pathways in human gut bacteria^46^. However, only enteropathogenic *Enterobacteriaceae*, including *S.* Typhimurium, can use 1,2-PDL and propionate as carbon sources to generate energy^47^. *S.* Typhimurium harnesses the 1,2-PDL and propionate catabolic pathways to convert those less-favored carbon sources into pyruvate and eventually ATP.

When *Salmonella* resides in an oxygen-abundant milieu, propionyl-CoA is integrated into the MCC, coupled with the citric acid cycle, and processed to release energy via an electron-transport chain that uses oxygen as the final electron acceptor. Meanwhile, in anaerobic conditions like the distal gut, *Salmonella* exploits the *cob, pdu*, and *prp* operons in concert with the *ttr* operon for anaerobic respiration using tetrathionate as the final electron acceptor instead^48^. The *cob-cbi* operon encodes factors required for vitamin B_12_ synthesis only under anaerobic conditions^49^. Ado-B_12_ is then used as a cofactor in the 1,2-PDL pathway to catabolize 1,2-PDL to propionyl-CoA in combination with propanol and propionate. Propionyl-CoA is further processed via the MCC and the citric acid cycle, as in aerobic conditions, whereas metabolic byproducts such as NADH and its equivalents are oxidized sequentially with tetrathionate as the electron acceptor. Tetrathionate, usually present in humid soils, has recently been found to be produced during the inflammation response to enteropathogenic infection. Hydrogen sulfide (H_2_S), produced by colonic bacteria, is detoxified to thiosulfate (S_2_O_3_^2−^) and further oxidized to tetrathionate (S_4_O_6_^2−^) by nitric oxide radicals and reactive oxygen species generated in the intestinal lumen^50^. In contrast to coliforms, which are inactivated by tetrathionate, *S.* Typhimurium possesses the *ttr* operon composed of *ttrABCRS*, which enables it to exploit tetrathionate as an electron acceptor in the respiratory chain. This process gives *S.* Typhimurium a competitive edge over gut microbiota and allows it to outgrow commensal bacteria and ultimately to achieve transmission to new recipients^51^,^52^.

Considering the opposite roles of EIIA^Ntr^ vis-à-vis the *cob*-*cbi, pdu*, and *prp* operons and SPI-1 and SPI-4 regulation, *S.* Typhimurium in an intestinal lumen enriched with 1,2-PDL and propionate seems to inevitably produce propionyl-CoA and thus lower the expression of SPI-1 and SPI-4, which are critical for *Salmonella* invasion into epithelial cells. Fine-tuning of *Salmonella* virulence by EIIA^Ntr^ has been reported in the regulation of SPI-2, a *Salmonella* pathogenicity island essential for the proliferation of *Salmonella* in macrophages^53^. *ssrA/ssrB*, which encode the two-component regulatory system of SPI-2, are expressed ectopically within phagosomes in response to intracellular cues. EIIA^Ntr^ directly interacts with SsrB, inhibiting SsrB from over activating the transcription of SPI-2 genes^14^. The differential roles of EIIA^Ntr^, which provides *S*. Typhimurium with a growth benefit over the competing microbiota but dampens invasion activity in the intestinal lumen abundant with 1,2-PDL and propionate, could be a sophisticated strategy for bacterial fitness inside the host. *S.* Typhimurium is a gastrointestinal pathovar that causes general inflammation in the human intestine but rarely disseminates into deeper tissues to cause systemic infection. However, invasive typhoidal *Salmonella* serovars such as *S.* typhi are prone to disrupt the intestinal epithelial barrier and spread over the whole body. Interestingly, the invasive *Salmonella* serovars evolutionally adapted for extraintestinal life are defective in anaerobic metabolic networks^54^. Bacterial functions attributable to the *cob, pdu, prp*, and *ttr* operons have been sequentially compromised during evolution away from an intestinal life. *S.* Typhimurium, adapted for intestinal life, might use EIIA^Ntr^ as a regulatory switch to balance between bacterial intestinal colonization and internalization into host cells in response to the environmental nutrient repertoire.

Thus, we conclude that EIIA^Ntr^ is a key player that not only controls *Salmonella* fitness in response to the availability of 1,2-PDL or propionate but also influences its internalization into host cells by modulating the production of a metabolic intermediate of propionyl-CoA during host infection.

## Methods

### Bacterial strains, media, and culture conditions

All bacterial strains were derived from *S.* Typhimurium SL1344 as the parent and are listed in Supplementary Table S3. Luria-Bertani (LB) medium containing 1% Bacto-tryptone, 0.5% yeast extract, and 1% NaCl (pH 7.5) was used as the complex culture medium for the routine growth of bacteria. All *S.* Typhimurium strains were grown aerobically at 37 °C with antibiotic supplementation at the following concentrations: ampicillin, 50 μg/ml; kanamycin, 50 μg/ml; chloramphenicol, 25 μg/ml. As an alternative carbon source, 1,2-PDL (12.5 mM) or propionate (10 mM) was added when bacterial cultures reached an optical density of 0.5–0.6 at 600 nm (OD_600_). In the case of 1,2-PDL utilization, Ado-B_12_ (20 nM) was also added as a cofactor to the LB medium.

### Construction of strains and plasmids

The *ptsN* deletion mutant strain was constructed using the lambda red recombination method for in-frame gene deletion^55^. For the construction of the Δ*ptsN* mutant strain, SR7001, the Km^R^ cassette from pKD13 was amplified using the primers ptsN-del-F and ptsN-del-R. The resulting PCR products were integrated into the *ptsN* region in the SL1344 wild-type strain containing the plasmid pKD46, followed by selection for Δ*ptsN*::kan transformants. The Km^R^ cassette was removed using the plasmid pCP20^55^. For the deletion of the *pdu* operon, the Cm^R^ cassette from pKD3 was amplified using the primers pduA~X-del-F and pduA~X-del-R. After that, the lambda red recombination method was followed, as mentioned above. The tagging of EIIA^Ntr^ and HilD with the FLAG and HA peptides, respectively, at the C-terminus was also performed using the same recombination system with each primer set: ptsN-FLAG-F/ptsN-FLAG-R and HilD-HA-F/HilD-HA-R. To construct pPtsN plasmid expressing *ptsN* gene, the *ptsN* gene was amplified by PCR using the primers ptsN-comple-F and ptsN-comple-R containing the restriction sites HindIII and SphI, respectively. The PCR product harboring the *ptsN* structural gene, its own promoter and RBS was introduced between the HindIII and SphI restriction sites of the pACYC184 vector. A new promoter was found at 67 base upstream of the ATG codon of the *ptsN* gene in addition to the one upstream of the *rpoN* gene in *S*. Typhimurium SL1344 (un-published data) anfd used for *ptsN* expression in pPtsN construction. To construct pPocR expressing PocR under the *lac* promoter, the *pocR* gene was amplified by PCR using the pocR-over-R and pocR-over-R primers, and the purified PCR product was inserted between the BamHI and HindIII sites of the pUHE21-2*lacI*^q^ vector^56^. Similarly, the *prpR* gene was amplified using the prpR-over-F and prpR-over-R primers, and then the PCR product was inserted into the pUHE21-2*lacI*^q^ vector via the BamHI and PstI sites to make pPrpR, which overexpresses PrpR. To construct the strain carrying a *lacZ* reporter gene, Km^R^ cassette from pKD13 was amplified using primer sets and the lambda red recombination method. Then the *lacZ* gene was introduced using the plasmid pCE70^57^. For *lacZ* fusion in mutant strains, the integrated *lacZ* gene was transferred using bacteriophage P22 transduction^58^. The sequences of primers used in constructing the *Salmonella* strains and plasmids are listed in Supplementary Table S4.

### RNA isolation and sequencing

*Salmonella* strains were grown in LB medium under aerobic conditions at 37 °C to the log phase with an OD_600_ of 1.2–1.4 (Supplementary Fig. S2). RNAprotect bacterial reagent (Qiagen, Hilden, Germany) was applied at an appropriate volume for each collected sample before total RNA extraction. Total RNA was isolated using the RNeasy mini kit (Qiagen) according to the manufacturer’s instructions, and residual DNA was removed using Ambion Turbo DNA-*free*^TM^ (ThermoFisher Scientific, Braunschweig, Germany). The quantity and quality of total RNA were examined using Agilent 2100 Bioanalyzers (Agilent Technologies, CA, USA), and the RNA integrity number (RIN) was determined. Only RNAs with a RIN > 9 were used in further experiments. The extracted total RNA was stored at −80 °C until use. All RNA-seq and alignment process were performed in Chunlab, Inc. (Seoul, Korea). Five micrograms of total RNA from each sample was used as starting material. If needed, the RNA was concentrated. The RNA was then subjected to subtractive hybridization/bead capture rRNA-removal using the Ribo-Zero kit (Epicentre Biotechnologies, WI, USA). The mRNA was fragmented ultrasonically and then converted into an RNA-seq library using the mRNA-seq library construction kit v.2 (Illumina, CA, USA) according to the manufacturer’s instructions. RNA 2 × 100 bp paired-end sequencing was performed using the Illumina GAII (Illumina) according to the manufacturer’s protocol (Supplementary Fig. S4). The genome sequence data of *S.* Typhimurium SL1344 for the reference genome was retrieved from the NCBI database. Quality-filtered reads were aligned with the reference genome using Bowtie2^59^. RNA-seq was carried out once for both wild-type and a Δ*ptsN* mutant strain. Gene expression was quantified as reads per kilobase per million mapped reads (RPKM)^60^. Differentially expressed genes (DEGs) with a fold change of 3 or more (p-value < 0.05) were filtered and visualized by the CLRNAseq program (Chunlab).

### Heat Map Generation

A heat map was drawn to analyze the global response pattern. The log_2_RPKM values of relative gene expression of the *Salmonella enterica* serovar Typhimurium SL1344 wild-type and Δ*ptsN* mutant strains were calculated. The heat map and hierarchical clusters were then generated using Gitools v2.2.2.

### Quantitative real-time RT-PCR

The total RNA samples were treated with RNase-free DNase (ThermoFisher Scientific), and cDNA was synthesized using RNA to cDNA EcoDry^TM^ Premix (random hexamers) (Clontech). Quantification of cDNA was carried out using 2 × iQ SYBR Green Supermix (Bio-Rad, CA, USA), and real-time amplification of the PCR products was performed using the iCycler iQ real-time detection system (Bio-Rad). The calculated threshold cycle (C_t_) corresponding to a target gene was normalized by the C_t_ of the control *gyrB* gene. The topoisomerase *gyrB* gene was chosen as a control because it showed no significant variation of *gyrB* expression. Experiments were performed in triplicate. The sequences of primers used in the quantitative reverse transcription-PCR (qRT-PCR) analysis are listed in Supplementary Table S5.

### Propanediol utilization assay

A neutral-red pH indicator was used to test for the formation of propionate^61^. The color of this indicator changes from red to yellow between pH 6.8 and 8.0. When the neutral-red was added to the LB media (0.0033% as a final concentration), the use of 1,2-PDL produced a red color due to the reduction in pH, indicating the formation of propionate. Broth colors were examined after 8 h incubation in LB media.

### β-Galactosidase assay

A *Salmonella* strain containing a *lacZ* gene was grown in LB medium with the indicated additives. β-Galactosidase assays were carried out in triplicate, and the activity was determined as described previously^62^.

### Western blot analysis

Proteins were separated by molecular weight using SDS-PAGE and were transferred to a polyvinylidene difluoride membrane. The membrane was blocked with 0.5% nonfat dry milk in 1× Tris-buffered saline-Tween 20 buffer and probed with anti-FLAG antibody (1:1,000 dilution, Sigma-Aldrich, Taufkirchen, Germany), anti-HA antibody (3:2,000 dilution, Sigma-Aldrich), or anti-DnaK antibody (1:10,000 dilution, Enzo Life Science, NY, USA) as primary antibodies. Anti-mouse IgG conjugated with peroxidase (3:5,000 dilution, Santa Cruz Biotechnology, CA, USA) was used as the secondary antibody in all western blot experiments. The chemiluminescent signals were developed with the West-Zol plus western blot detection system (iNtRON Biotechnology, Seongnam, Korea).

### Analysis of EIIA^Ntr^ and HilD stability

Protein stability was determined using a previously described method^63^. *Salmonella* strains encoding EIIA^Ntr^-FLAG or HilD-HA protein from the chromosome were grown in LB medium with the indicated additives for 4 h, and chloramphenicol was added at 0.2 mg/ml to block *de novo* protein synthesis. Aliquots of those cultures were collected at the indicated time points after the addition of chloramphenicol and were subjected to western blotting, as described above.

### Gentamicin protection assay

Caco-2 cells were grown in Dulbecco modified Eagle medium (DMEM) supplemented with 10% heat-inactivated fetal bovine serum (FBS), penicillin (50 U/ml), and streptomycin (50 U/ml). At least 1 h before bacterial infection, a monolayer of 2.5 × 10^5^ Caco-2 cells was prepared in a 24-well tissue culture plate and incubated in DMEM-10% FBS without antibiotics at 37 °C under 5% CO_2_. Bacteria were subcultured from an overnight culture (1:100) in fresh LB medium containing the indicated additives and grown for 4 h at 37 °C with aeration. Bacteria were applied to the cell monolayer at a multiplicity of infection (MOI) of 10. After 30 min of incubation at 37 °C under 5% CO_2_, non-invasive bacteria were removed by washing three times with pre-warmed phosphate-buffered saline (PBS) and were then incubated for 1 h with the pre-warmed medium supplemented with 100 μg/ml of gentamicin to kill extracellular bacteria. Afterward, the wells were washed three times with pre-warmed PBS, lysed in 1% Triton X-100 in PBS for 30 min at 37 °C, and then diluted in PBS. A dilution of the suspension was plated on LB agar medium to enumerate the CFU.

## Additional Information

**How to cite this article:** Yoo, W. *et al*. Enzyme IIA^Ntr^ regulates *Salmonella* invasion via 1,2-propanediol and propionate catabolism. *Sci. Rep.* **7**, 44827; doi: 10.1038/srep44827 (2017).

**Publisher's note:** Springer Nature remains neutral with regard to jurisdictional claims in published maps and institutional affiliations.

## Supplementary Material

Supplementary Information
