# Supplementary material for: Enzyme IIANtr Regulates Salmonella Invasion Via 1,2-Propanediol And Propionate Catabolism
Source: Sci Rep. 2017 Mar 23;7:44827. doi: 10.1038/srep44827 (PMC5363084; doi:10.1038/srep44827)
Supplement: Supplementary Information [file srep44827-s1.pdf]

Supplementary information

Enzyme IIA^{Ntr} regulates *Salmonella* invasion via 1,2-propanediol and propionate catabolism

Woongjae Yoo[1,†], Dajeong Kim[1,†], Hyunjin Yoon[2], Sangryeol Ryu[1,3,*]

[1]Department of Food and Animal Biotechnology, Department of Agricultural Biotechnology, and Research Institute for Agriculture and Life Sciences, Seoul National University, Seoul 08826, Korea.

[2]Department of Molecular Science and Technology, Department of Applied Chemistry and Biological Engineering, Ajou University, Suwon 16499, Korea.

[3]Center for Food and Bioconvergence, Seoul National University, Seoul 08826, Korea.

*Correspondence and requests for materials should be addressed to S.R. (email: sangryue@snu.ac.kr)

[†]These authors contributed equally to this work.

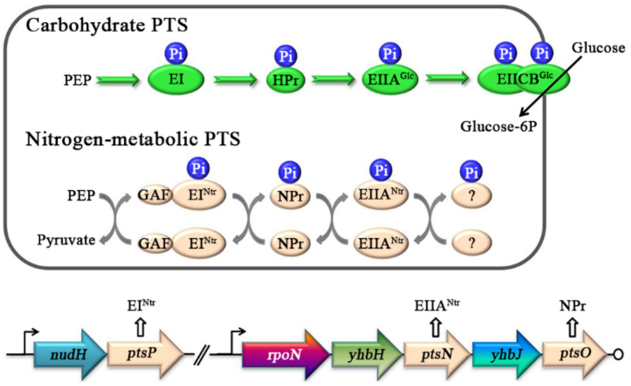

Figure S1. Organization of the nitrogen-PTS in *Salmonella* Typhimurium. The nitrogen-PTS catalyzes a cascade of phosphoryl-transfer reactions. First, EI^{Ntr} autophosphorylates with phosphoenolpyruvate and subsequently transfers the phosphoryl group to NPr, which then phosphorylates $EIIA^{Ntr}$. The final acceptor for the phosphoryl groups of $EIIA^{Ntr}$ remains unknown. NPr and $EIIA^{Ntr}$ are encoded in the *rpoN* operon. EI^{Ntr} is encoded elsewhere on the chromosome in the *nudH-ptsP* operon.

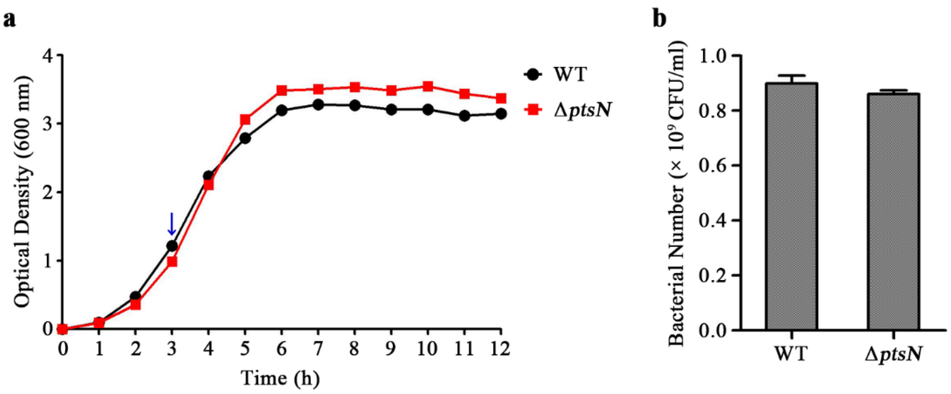

Figure S2. No significant difference was found in bacterial growth rate or viability between *Salmonella* Typhimurium SL1344 wild-type and the Δ*ptsN* mutant strain. (A) Overnight cultures of *Salmonella* Typhimurium SL1344 wild-type and Δ*ptsN* mutant strain were harvested at 13,000 × g for 2 min, and the cells were resuspended in fresh LB medium and incubated at 37°C with shaking. The OD_{600} value was measured every 1 h to draw and compare the growth curve. This experiment was performed in triplicate. (B) To compare the bacterial cell viability between the wild-type and Δ*ptsN* mutant strains, cultures were collected at 3 h (blue arrow, Fig. S3A) and then serially diluted in PBS. Dilutions of the suspension were plated on LB agar medium to enumerate the CFU. This experiment was performed in triplicate.

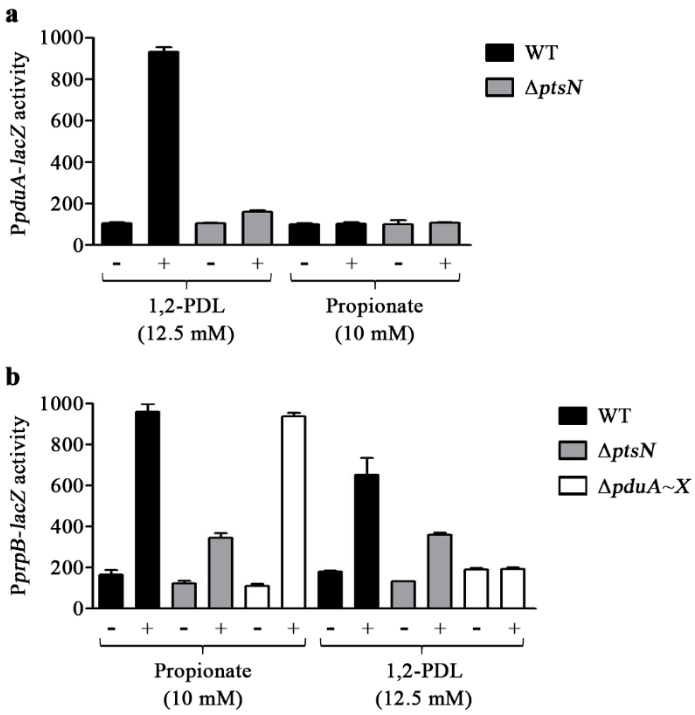

Figure S3. The effects of *ptsN* on the expression of *pduA* and *prpB* genes in the presence of 1,2-PDL or propionate. (A) The effects of *ptsN* mutation on the *pduA* gene measured by a β-galactosidase assay using a P*pduA*-*lacZ* reporter in the wild-type and Δ*ptsN* mutant strains. These strains were cultivated in LB medium in the presence or absence of either 1,2-PDL or propionate. (B) Transcriptional expression of the *prpB* gene, also measured by a β-galactosidase assay using a P*prpB*-*lacZ* reporter in the wild-type, Δ*ptsN*, and Δ*pduA~X* strains. These were grown in LB medium in the presence or absence of either 1,2-PDL or propionate.

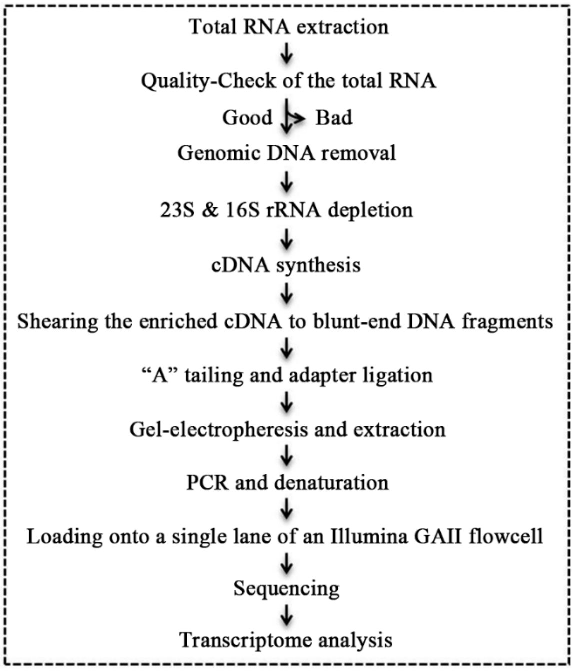

Figure S4. The whole process of RNA sequencing. After the quality-check of total RNA, rRNA was depleted, and mRNA was converted into cDNA. The synthesized cDNA was sheared to blunt-ends and ligated with adapter containing primer sites for sequencing. Adequate cDNA fragments were selected via gel-electrophoresis and then denatured to form a single strand after PCR. The completed samples were loaded onto a single lane of an Illumina GAII flow cell and sequenced. Finally, transcriptome analysis was conducted.

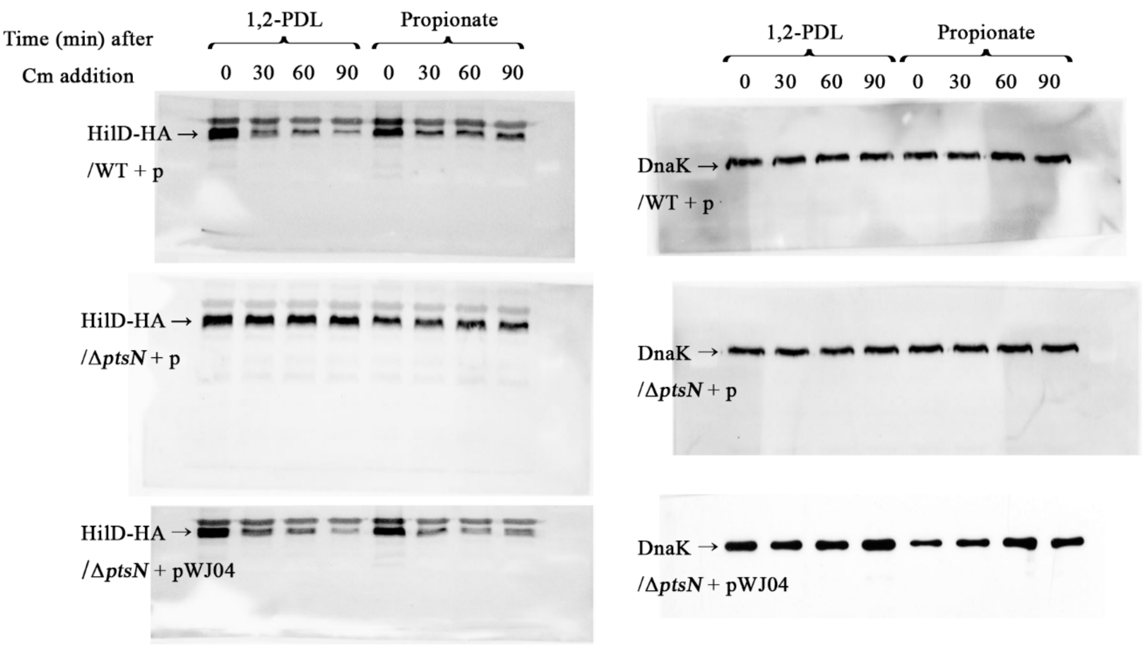

Figure S5. Full-length blots of Figure 3d and 3e. Equivalent amounts of proteins were subjected to SDS-PAGE analysis and transferred to PVDF membranes. PVDF membranes were cut into two pieces to detect HilD-HA and DnaK, respectively. The blots of target proteins were indicated with an arrow.

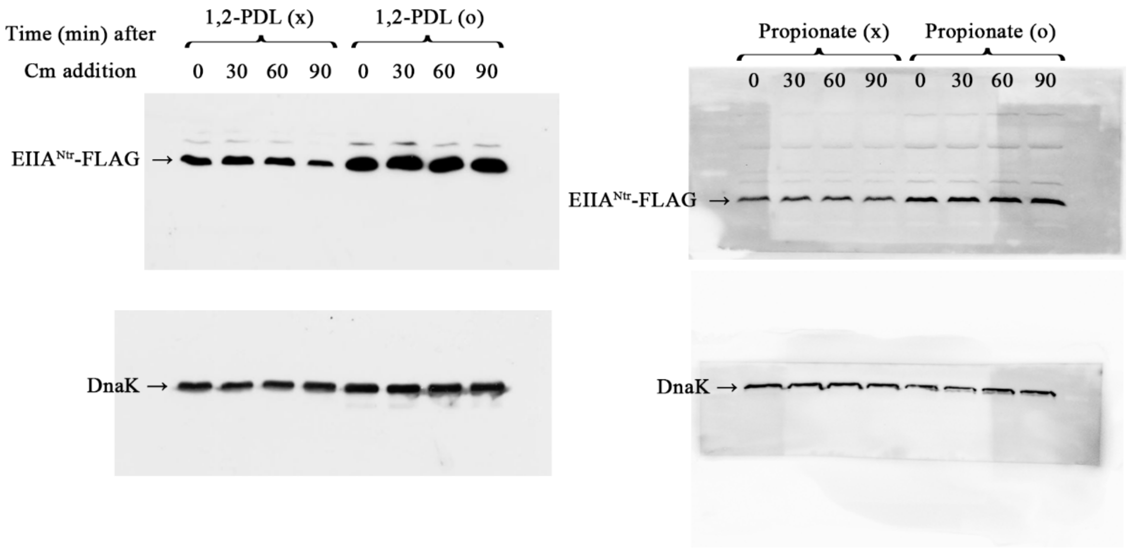

Figure S6. Full-length blots of Figure 6c and 6d. Equivalent amounts of proteins were subjected to SDS-PAGE analysis and transferred to PVDF membranes. PVDF membranes were cut into two pieces to detect EIIA^{Ntr}-FLAG and DnaK, respectively. The blots of target proteins were indicated with an arrow.

**TABLE S1 Summary of RNA-seq and data analysis results.**				
	**wild-type**		**Δ*ptsN***	
	number	% of total	number	% of total
Total number of reads	22,388,642	100	14,255,406	100
Mapped reads	22,293,048	99.6	14,163,120	99.4
-uniquely mapped	21,285,343	95.1	13,820,149	97.0
-non-specifically mapped	1,007,705	4.5	342,971	2.4
Unmapped reads	95,594	0.4	92,286	0.6

**TABLE S2 Genes specifically regulated by *ptsN*.**			
**Category**	**Gene**	**Fold expression**	**Function**
Propionate catabolism	*prpR*	-1.95	propionate catabolism operon regulatory protein
	prpB	-1.86	carboxyvinyl-carboxyphosphonate phosphorylmutase
	prpC	-0.68	methylcitrate synthase
	prpD	-2.82	PrpD protein
	prpE	-1.14	PrpE protein
Vitamin B_{12} synthesis	*cobC*	0.18	alpha-ribazole-5'-phosphate phosphatase
	cobD	-0.80	putative aminotransferase CobD
	cobB	0.16	NAD-dependent deacetylase
	cobT	-1.37	nicotinate-nucleotide-dimethylbenzimidazole phosphoribosyltransferase
	cobS	-0.91	cobalamin (5'-phosphate) synthase
	cobU	-0.37	cobinamide kinase and guanylyltransferase
	cbiP	-1.18	putative cobyric acid synthase
	cbiO	-0.88	putative cobalt transport ATP-binding protein
	cbiQ	-0.79	putative cobalt transport protein
	cbiN	-0.34	putative cobalt transport protein CbiN
	cbiM	-0.43	cobalamin biosynthesis protein
	cbiL	-0.81	cobalt-precorrin-2 C(20)-methyltransferase

	cbiK	-0.41	sirohydrochlorin cobaltochelatase
	cbiJ	-0.78	cobalt-precorrin-6a reductase
	cbiH	-0.85	precorrin-3 C17-methyltransferase
	cbiG	-0.88	cobalamin biosynthesis protein
	cbiF	-0.84	precorrin-4 C(11)-methyltransferase
	cbiT	-0.68	precorrin-8W decarboxylase
	cbiE	-0.63	precorrin-6Y C5,15-methyltransferase
	cbiD	-1.04	cobalt-precorrin-6A synthase
	cbiC	-1.41	precorrin-8X methylmutase
	cbiB	-0.76	cobalamin biosynthesis protein
	cbiA	-0.62	cobyrinic acid A,C-diamide synthase
1,2-PDL utilization	*pocR*	-0.25	pdu/cob regulatory protein
	pduF	-1.97	putative propanediol diffusion facilitator
	pduA	-2.43	putative propanediol utilization protein
	pduB	-3.86	putative propanediol utilization protein
	pduC	-3.57	propanediol dehydratase large subunit
	pduD	-4.03	propanediol dehydratase medium subunit
	pduE	-3.86	propanediol dehydratase small subunit
	pduG	-4.34	putative propanediol utilization
	pduH	-4.58	putative propanediol utilization
	pduJ	-3.68	putative propanediol utilization
	pduK	-3.70	putative propanediol utilization

	pduL	-4.07	putative propanediol utilization
	pduM	-3.76	putative propanediol utilization
	pduN	-5.90	putative propanediol utilization
	pduO	-5.40	propanediol utilization protein
	pduP	-3.36	putative propionaldehyde dehydrogenase
	pduQ	-4.33	putative propanediol dehydrogenase
	pduS	-3.62	propanediol utilization ferredoxin
	pduT	-3.50	putative propanediol utilization protein
	pduU	-1.00	putative propanediol utilization protein PduU
	pduV	-31.40	putative propanediol utilization protein PduV
	pduW	-2.89	acetokinase
Salmonella pathogenicity island-1	*hilC*	0.89	AraC family transcriptional regulator
	orgC	1.88	hypothetical protein
	orgB	1.46	oxygen-regulated invasion protein
	orgA	1.77	oxygen-regulated invasion protein
	prgK	1.75	type III secretion system apparatus
	prgJ	1.53	type III secretion system apparatus
	prgI	1.67	type III secretion system apparatus
	prgH	1.65	type III secretion apparatus component
	hilD	1.43	AraC family transcriptional regulator
	hilA	1.04	invasion protein regulator

	iagB	1.16	cell invasion protein
	sptP	1.76	tyrosine phosphatase (associated with virulence)
	sicP	2.11	chaperone (associated with virulence)
	iacP	2.73	acyl carrier protein
	sipA	1.92	pathogenicity island 1 effector protein
	sipD	1.88	pathogenicity island 1 effector protein
	sipC	2.11	pathogenicity island 1 effector protein
	sipB	1.83	pathogenicity island 1 effector protein
	sicA	1.95	type III secretion-associated chaperon
	spaS	1.63	type III secretion system secretory apparatus
	spaR	1.33	type III secretion system secretory apparatus
	spaQ	0.83	type III secretion system secretory apparatus
	spaP	1.74	type III secretion system secretory apparatus
	spaO	1.06	surface presentation of antigens protein (associated with type III secretion and virulence)
	invJ	0.93	surface presentation of antigens protein (associated with type III secretion and virulence)
	invI	1.77	type III secretion system secretory apparatus
	invC	1.14	secretory apparatus ATP synthase (associated with virulence)
	invB	1.49	chaperone protein for type III secretion system effectors
	invA	1.69	secretory apparatus of type III secretion system
	invE	1.38	cell invasion protein
	invG	1.27	type III secretion system secretory apparatus
	invF	1.53	AraC family regulatory protein

	invH	1.80	outer membrane lipoprotein
Salmonella pathogenicity island-4	*siiA*	2.29	type I secretion-related protein
	siiB	2.50	putative integral membrane protein
	siiC	3.06	putative type I secretion protein
	siiD	3.19	putative type I secretion protein
	siiE	2.50	large repetitive protein
	siiF	3.16	putative type-1 secretion protein

**TABLE S3 Bacterial strains and plasmids used in this study.**		
Strains	**Description**	**Reference or source**
***Salmonella enterica* serovar Typhimurium**		
SL1344	Wild type, Sm^R	1
SR7001	Δ*ptsN*	2
SR7021	$EIIA^{Ntr}$-FLAG	2
SR7024	HilD-HA	This study
SR7025	HilD-HA, Δ*ptsN*	This study
SR7026	P*ptsN-lacZ*	This study
SR7027	P*prpB-lacZ*	This study
SR7028	P*prpB-lacZ*, Δ*ptsN*	This study
SR7029	P*prpB-lacZ*, Δ*pduABCDEGHJKLMNOPQSTUVWX*	This study
SR7030	P*pduA-lacZ*	This study
SR7031	P*pduA-lacZ*, Δ*ptsN*	This study
Escherichia coli		
DH5α	*gyrA96 recA1 relA1 endA1 thi-1 hsdR17 glnV44 deoR Δ(lacZYA-argF)U169* [Φ80d Δ(*lacZ*)*M15*]	3
**Plasmids**		
pKD46	Ap^R P_{BAD}-*gam-beta-exo oriR101 repA101^{ts}*	4
pKD13	Ap^R FRT Km^R FRT PS1 PS4 *oriR6Kγ*	4
pCP20	Ap^R Cm^R *cI*857 λP_R*flp oripSC101^{ts}*	4

pCE70	Km^R FRT *tnpR lacZY*$^+$ *oriR6Kγ*	5
pACYC184	Tet^R Cm^R p15A *ori*	6
pPtsN	pACYC184-*ptsN*	2
pPocR	pUHE21-2*lacI*^{q}-*pocR*	This study
pPrpR	pUHE21-2*lacI*^{q}-*prpR*	This study

TABLE S4 Primers used for the construction of bacterial strains and plasmids.	
**Primers**	**Sequences (5' to 3')**
ptsN-del-F	CTG GCC ATC AAC CTG ACA GGA CAG GTT CTT AGG TGA AAT TTG TAG GCT GGA GCT GCT TCG
ptsN-del-R	CGT TTC GCC ACC AGC GAC AGC GTG TGC AGA TGC GTT TTA ATT CCG GGG CTC CGT CGA CC
ptsN-comple-F	AAA AAG CTT ACG CAC ATC TCG GAT GCG AC
ptsN-comple-R	AAA GCA TGC CCG CTG ACG ATC ATC AGT AC
hilD-HA-F	TAA AAC TAC GCC ATC GAC ATT CAT AAA AAT GGC GAA CCA TTA TCC GTA TGA TGT TCC TGA TTA TGC TAG CCT CTA ATG TAG GCT GGA GCT GCT TCG
hilD-HA-R	TTA ATA AAA ATC TTT ACT TAA GTG ACA GAT ACA AAA AAT GAT TCC GGG GAT CCG TCG ACC
ptsN-FLAG-F	TCA AAT CAT TAC TGA CAC CGA AGG TGA GCA GAA TGA GGC AGG CAG CGG CGA CTA CAA AGA CGA TGA CGA CAA GTA ATG TAG GCT GGA GCT GCT TCG
ptsN-FLAG-R	GTT TCT CCT CAC AAC GAC AGA AAT AAA TGC CAT TGA GTT GAT TCC GGG GAT CCG TCG ACC
ptsN-lacZ-F1	AAT CAT TAC TGA CAC CGA AGG TGA GCA GAA TGA GGC ATA ATG TAG GCT GGA GCT GCT TCG
ptsN-lacZ-R1	ACC ATG TAC CGT TTC TCC TCA CAA CGA CAG AAA TAA ATG CAT TCC GGG GAT CCG TCG ACC
pduA-lacZ-F1	CGA TGT AGA AAA AAT CTT ACC GAA GGG AAT TAG CCA ATG ATG TAG GCT GGA GCT GCT TCG

pduA-lacZ-R1	CAC ACG GGC AAT CAC CTG CGC CAT GAT CTG TTC CAC CAG CAT TCC GGG GAT CCG TCG ACC
prpB-lacZ-F1	CCC AAC GGG CCG GGT ATC AGG CTA TCT ATC TTT CTG GCG GGT GTA GGC TGG AGC TGC TTC
prpB-lacZ-R1	AAT CCC CAG ATC CGG CAG TCC GAG CGA GCC CGC CGC TAC GCT GTC AAA CAT GAG AAT TAA
pduA~X-del-F	AGC CGG ATA AGC CGC GTT CGG CAA CGG GTT AGA CTC AGC GGT GTA GGC TGG AGC TGC TTC
pduA~X-del-R	CGC GCT GCC GCC CTT TCC CGA CTT TAC ACC CCA CTA TTC AAT GGG AAT TAG CCA TGG TCC
pocR-over-F	AAA GGA TCC TTT CAC GCC GTT TTG TCA GT
pocR-over-R	AAA AAG CTT ACT ATC AAA AAT CGG CAA TAG C
prpR-over-F	AAA GGA TCC ACG ACT GCC CAC AGC GCT
prpR-over-R	AAA CTG CAG TTG TCT TAA TTA TCC GAC TGG TC

TABLE S5 Primers used in qRT-PCR analysis.	
**Primers**	**Sequences (5' to 3')**
qRT-copT-F1	TCT GAT CCC GTC GCA CTT TT
qRT-copT-R1	CCT CCG GCA GGA CAA TAT TAC T
qRT-cbiA-F1	CCC ACT ATC AGC TCC TCA AA
qRT-cbiA-R1	CAC ATC CAC TGT TTG TTC CA
qRT-pocR-F1	TTT CAC GCC GTT TTG TCA GTT
qRT-pocR-R1	GAA CCT GCC CGC ATA AAA CA
qRT-pduF-F1	GCG GTA GCG TCG AAA GTT TG
qRT-pduF-R1	AAC GGC AAC CAG TAT CCC AAT
qRT-pduA-F1	GGA ATG GTA GAA ACC AAA GGC TTA
qRT-pduA-R1	GCC AAT CTT TTC ATA GCC CAC TA
qRT-pduC-F1	GTT CGC CCA GGA AAT CAT CA
qRT-pduC-R1	ATA ATG GCG GAG GTG TGC AA
qRT-pduP-F1	AAC CCG ACG GAA ACC ATC AT
qRT-pduP-R1	ATC TGC TGG GTC GCT TCG AA
qRT-pduW-F1	GGC TGA TTG AGC GTA TCG GTA T
qRT-pduW-R1	CGT TCG ATT TGC GCT AAC G
qRT-ptsN-F1	AGC GGC GTT CAT TGT CAG A
qRT-ptsN-R1	GGA GGT AAA CTG AGC TGT TTT GC
qRT-hilD-F1	CAA CGA CTT GGC GCT CTC TAT
qRT-hilD-R1	ACA GGA GAA CGC CGT TTT CA
qRT-hilA-F1	CTG CCG GTG ACC ATT ACG A

qRT-hilA-R1	GCG GGT TGG TGT TCT ATC AAC T
qRT-invF-F1	GGG TTT TGC TGA GTC CTG AGT T
qRT-invF-R1	TTC TCC CAG CAT TCT CAT CGT
qRT-spaS-F1	CCG CTA AAA AAG GCC AGT CA
qRT-spaS-R1	AAA TGA GCC ATA CGA CAC CAG AT
qRT-sopE2-F1	CCA CCC AGC ACT ACA GAA TCC
qRT-sopE2-R1	GAT GCA GGC TAA AAC GAT CTG A
qRT-sipA-F1	CCA ACG CTT CAT CCG AAA GT
qRT-sipA-R1	AAG CGG CTT CAC ATT CAC AAT
qRT-siiA-F1	ATG GCA GGC TGA GAA GCT TTT
qRT-siiA-R1	CCC GTT CAT ATC CCA GTC GTA
qRT-siiE-F1	TGG TTG CCA GCG TTG ATG T
qRT-siiE-R1	GCT CAA TCG TCG GCT TTT CT
qRT-gyrB-F1	ATA ACG CCA CGC AGA AAA TGA
qRT-gyrB-R1	TGG CTG ATA CAC CAG CTC TTT G
qRT-prpR-F1	CGG CTT TAC TTG CCT TTC AG
qRT-prpR-R1	TGT CAG ACG GGT CAT ATC CA
qRT-prpB-F1	GGG ATT TCT ACG CTG GAT GA
qRT-prpB-R1	CCA TCT CCT CTT TCG AGA C
qRT-prpC-F1	TTC AAC GCC TCG ACG TTT AC
qRT-prpC-R1	CCA AAG CCA ATC ACC ACC TC
qRT-prpD-F1	TCC GGT ACA GGC AGC TTT TA
qRT-prpD-R1	CGA TGC AGC CCT GAA TTT CA

qRT-prpE-F1	TTT ACA CCT CCG GCA CTA CC
qRT-prpE-R1	GGC GCG TAA ACG ATA TAG GA

REFERENCES

1. Lucas, R. L. & Lee, C. A. Unravelling the mysteries of virulence gene regulation in *Salmonella* Typhimurium. *Mol. Microbiol.* **36**, 1024-1033 (2000).
2. Yoo, W. *et al.* Fine-tuning of amino sugar homeostasis by $EIIA^{Ntr}$ in *Salmonella* Typhimurium. *Sci. Rep.* **6**, 33055 (2016).
3. Woodcock, D. *et al.* Quantitative evaluation of *Escherichia coli* host strains for tolerance to cytosine methylation in plasmid and phage recombinants. *Nucleic Acids Res.* **17**, 3469-3478 (1989).
4. Datsenko, K. A. & Wanner, B. L. One-step inactivation of chromosomal genes in *Escherichia coli* K-12 using PCR products. *Proc. Natl. Acad. Sci. USA* **97**, 6640-6645 (2000).
5. Merighi, M., Ellermeier, C. D., Slauch, J. M. & Gunn, J. S. Resolvase-in vivo expression technology analysis of the *Salmonella enterica* serovar Typhimurium PhoP and PmrA regulons in BALB/c mice. *J. Bacteriol.* **187**, 7407-7416 (2005).
6. Chang, A. C. & Cohen, S. N. Construction and characterization of amplifiable multicopy DNA cloning vehicles derived from the P15A cryptic miniplasmid. *J. Bacteriol.* **134**, 1141-1156 (1978).
